# Effects of Intracellular Calcium and Actin Cytoskeleton on TCR Mobility Measured by Fluorescence Recovery

**DOI:** 10.1371/journal.pone.0003913

**Published:** 2008-12-11

**Authors:** Omer Dushek, Sabina Mueller, Sebastien Soubies, David Depoil, Iris Caramalho, Daniel Coombs, Salvatore Valitutti

**Affiliations:** 1 Department of Mathematics and Institute of Applied Mathematics, University of British Columbia, Vancouver, British Columbia, Canada; 2 INSERM, U563, Centre de Physiopathologie de Toulouse Purpan, Section Dynamique moléculaire des interactions lymphocytaires, Toulouse, France; New York University School of Medicine, United States of America

## Abstract

**Background:**

The activation of T lymphocytes by specific antigen is accompanied by the formation of a specialized signaling region termed the immunological synapse, characterized by the clustering and segregation of surface molecules and, in particular, by T cell receptor (TCR) clustering.

**Methodology/Principal Findings:**

To better understand TCR motion during cellular activation, we used confocal microscopy and photo-bleaching recovery techniques to investigate the lateral mobility of TCR on the surface of human T lymphocytes under various pharmacological treatments. Using drugs that cause an increase in intracellular calcium, we observed a decrease in TCR mobility that was dependent on a functional actin cytoskeleton. In parallel experiments measurement of filamentous actin by FACS analysis showed that raising intracellular calcium also causes increased polymerization of the actin cytoskeleton. These *in vitro* results were analyzed using a mathematical model that revealed effective binding parameters between TCR and the actin cytoskeleton.

**Conclusion/Significance:**

We propose, based on our results, that increase in intracellular calcium levels leads to actin polymerization and increases TCR/cytoskeleton interactions that reduce the overall mobility of the TCR. In a physiological setting, this may contribute to TCR re-positioning at the immunological synapse.

## Introduction

The activation of T lymphocytes by antigenic ligands displayed on the surface of antigen presenting cells (APC) is the central event in developing an adaptive immune response. During the T cell/APC interaction, TCR bind to peptide-major-histocompatibility-complex (pMHC) present on the surface of the APC [1]. T cells are observed to respond sensitively and specifically to antigenic stimulation [2], [3].

Upon conjugation between T cells and APC, TCR and accessory molecules move towards the T cell/APC contact site, forming a signaling area at the interface that has been named the immunological synapse (IS) [4], [5]. TCR movement towards the IS has been thoroughly documented and shown to reflect the intensity of antigenic stimulation [6], [7] but we do not have a complete picture of how surface receptor signaling modulates TCR motion.

TCR motion on live cells has been studied using fluorescence recovery after photo-bleaching (FRAP, [8]) and fluorescence loss in photo-bleaching (FLIP, [8]) techniques. In a primary study, Sloan-Lancaster et al. showed that GFP-tagged CD25/ζ-chain chimeras expressed on the surface of HeLa cells exhibit very low mobility [8]. Following this observation, we employed a TCR β chain-deficient Jurkat T cell line transfected with a GFP/fusion TCR β-chain to measure lateral mobility of fully assembled TCR/CD3 complexes using FRAP. We showed that TCR are mobile on the surface of Jurkat T cells [9]. Using a different approach, M. Krummel and colleagues also showed that TCR are mobile on the surface of murine T cells and that they accelerate towards the IS during antigen recognition [10]. More recently, it has been shown that TCR dynamically form micrometer-scale clusters that move in a directed fashion towards the center of the IS during antigen recognition [11]–[13]. TCR recruitment to the center of the IS has been associated with productive signaling [5], [14] and with TCR internalization [13], [15].

TCR directed motion requires an energy-consuming mechanism and a natural candidate for this mechanism is an interaction between TCR and the cortical actin cytoskeleton (CAC). T cells need a functional actin cytoskeleton to form productive conjugates with APC [13], [16], [17] and cortical actin has further been implicated in the transport of IS components to the T cell/APC contact site [18]–[20]. However, an explicit link between the actin cytoskeleton and TCR dynamics has not been established at the molecular level. In particular it is not clear how activation signals triggered by TCR engagement might modulate TCR mobility via CAC-dependent mechanisms.

Here, we report on efforts to characterize TCR mobility in human T lymphocytes using fluorescence recovery techniques. We stimulated T cells using ionomycin, a drug known to increase the concentration of intracellular calcium ([Ca^2+^]_i_). We show that [Ca^2+^]_i_ increase markedly reduces TCR mobility on the T cell surface via an actin cytoskeleton-dependent mechanism. We also show that actin polymerization increases following [Ca^2+^]_i_ increase, suggesting a direct link between calcium induced CAC polymerization and constraints on TCR mobility on the T cell surface.

## Results

### TCR mobility is similar in naïve and activated human T cells

**Figure 1:**
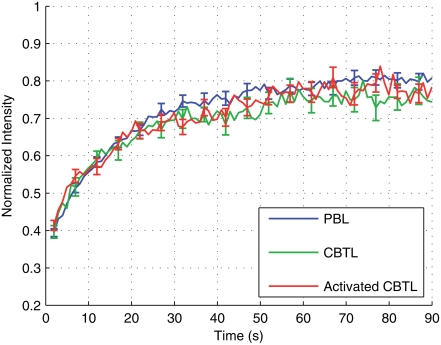
TCR mobility does not depend on the activation state of T cells. Mean FRAP recovery curves are shown for peripheral blood T Lymphocytes (PBL, blue curve n = 61), Cord Blood T Lymphocytes either freshly isolated (CBTL, green curve n = 20) or activated in culture (activated CBTL, red curve n = 20). Data are from 2 (for CBTL) and 4 (for PBL) independent experiments.

We initially focused on studying TCR mobility on unstimulated T cells. We used three CD4^+^ T cell populations: naïve T lymphocytes freshly isolated from cord blood (CBTL); T cells from a line obtained by cultivating for two weeks *in vitro* naïve T cells in the presence of magnetic beads coated with anti-CD3 plus anti-CD28 mAb (activated CBTL); freshly isolated T lymphocytes from peripheral blood of adult healthy donors (PBL). We stained cells from each population using anti-CD3 Fab antibodies labeled with Cy5 [7], [21] and performed FRAP experiments. We applied the method described in Dushek at al. [22] to analyze FRAP recovery curves and extract the mobility parameters of the TCR. Two parameters were calculated: the diffusion coefficient (D, generally expressed in µm^2^/s) and the mobile fraction (M_f_), indicating the fraction of TCR that are mobile on the timescale of the experiment. The results of this analysis are presented in Figure 1 and Table 1. TCR had a diffusion coefficient (D, expressed as mean±SEM)) of 0.048±0.008 µm^2^/s in CBTL, 0.035±0.006 µm^2^/s in activated CBTL and 0.061±0.008 µm^2^/s in PBL. The values of D were not found to be significantly different (p>0.05). The mobile fraction (M_f_) of TCR was very high in all cell populations, supporting the notion that TCR are constitutively mobile on living T cells (Table 1).

**Table 1:** Diffusion coefficient and mobile fraction under various pharmacological treatments.

Condition	n	Diffusion Coefficient	Mobile Fraction	P-Value of Diffusion
		(µm^2^/s)	(M_f_)	Relative to PBL*
PBL	61	0.061±0.008	0.96±0.04	1.00
CBTL	20	0.048±0.008	1.00±0.05	0.40
Activated CBTL	20	0.035±0.006	1.09±0.04	0.08
PBL+CytoD	20	0.068±0.014	0.93±0.05	0.66
PBL+LatB	10	0.082±0.016	0.95±0.06	0.34
PBL+Iono	37	0.017±0.002	1.00±0.04	0.0001
PBL+Iono+CytoD	25	0.043±0.005	0.92±0.04	0.17
PBL+Iono+LatB	10	0.045±0.009	0.99±0.05	0.45

* P-values were obtained by comparing diffusion coefficients from each experiment to the untreated PBL data using a two-sample T-test. Except for PBL+Iono no significant differences in the diffusion coefficient were observed (P-values>0.05) No significant differences in the mobile fraction were observed (all P-values>0.05).

These results show that the basic parameters of TCR dynamics on the surface of T cells are not significantly different in T cells at different stages of activation, indicating that TCR mobility is not involved in allowing activated T cells to be more responsive to antigenic stimulation.

### [Ca^2+^]_i_ increase affects TCR mobility by an actin cytoskeleton dependent mechanism

**Figure 2:**
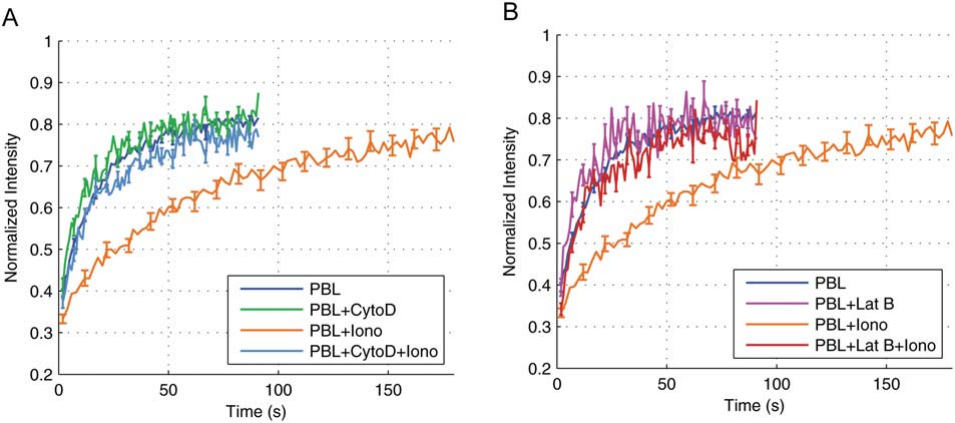
TCR mobility is modulated by intracellular calcium via the actin cytoskeleton. A. Mean FRAP recovery curves are shown for Peripheral blood T Lymphocytes (PBL, blue curve n = 61), PBL treated with cytochalasin D (PBL+CytoD, green curve n = 20), PBL treated with ionomycin (PBL+iono, orange curve n = 37), and PBL treated with ionomycin and cytochalasin D (PBL+Iono+CytoD, light blue curve n = 25). B. Mean FRAP recovery curves are shown for PBL (blue line, n = 61) PBL treated with latrunculin B (PBL+LatB, pink curve n = 10), PBL treated with ionomycin (PBL+iono, orange curve n = 37), and PBL treated with ionomycin and latrunculin B (PBL+Iono+LatB, red curve n = 10).

We next investigated whether calcium signaling in T cells could affect TCR mobility. To this end, T cells were loaded with the calcium probe FLUO-4 (a dye that increases its light emission when bound to Ca^2+^) before staining with anti-CD3/Cy5 Fab antibodies. This allowed us to detect signal transduction in parallel with TCR dynamics [7], [23]. We stimulated T cells with ionomycin to mimic the [Ca^2+^]_i_ increase that follows productive TCR engagement [24]. Our results show that [Ca^2+^]_i_ increase results in a substantial decrease in TCR lateral diffusion in PBL, as measured by a reduction of D (Fig. 2A and Table 1). Pre-treatment of T cells with 10 µM cytochalasin D (a drug that inhibits actin cytoskeleton function) abolished the effect of ionomycin on TCR mobility (Fig. 2A). The values of D were 0.017±0.002 µm^2^/s in PBL treated with ionomycin, 0.068±0.014 µm^2^/s in PBL treated with cytochalasin D only and 0.043±0.004 µm^2^/s in PBL treated with both cytochalasin D and ionomycin. Similar results were obtained when T cells were treated with 50 nM latrunculin B instead of cytochalasin D (Fig. 2B). Interestingly, cytochalasin D/latrunculin B treatements did not significantly affect basal lateral mobility of TCR indicating that this process is not dependent on actin cytoskeleton function. Ionomycin treatment, although affecting TCR lateral diffusion (D values) did not affect the mobile fraction of TCR that was again close to 1 in the different samples (Table 1). Our results indicate that the activation of the calcium signaling pathway reduces TCR mobility in T cells by an actin cytoskeleton-dependent mechanism. They also show that [Ca^2+^]_i_ increase, although reducing the mobility of individual TCR, does not reduce the number of mobile molecules.

### [Ca^2+^]_i_ increase induces polymerization of the actin cytoskeleton

**Figure 3:**
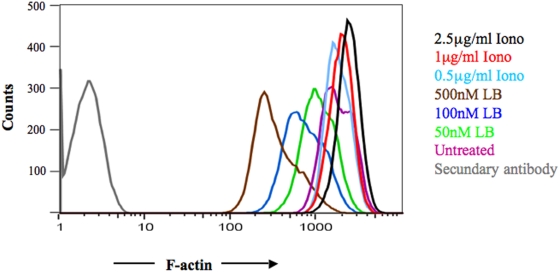
[Ca^2+^]_i_ increase induces actin polymerization in PBL. PBL were treated for 15 minutes with latrunculin B (50 nM, 100 nM or 500 nM) or with ionomycin (0.5 µg/ml, 1 µg/ml, 2.5 µg/ml). F-actin cellular content was measured by FACS analysis. Data are from one representative experiment out of three. In parallel experiments the vehicle of the drugs (DMSO) did not affect actin polymerization.

The above results suggested that, following [Ca^2+^]_i_ increase, the actin cytoskeleton becomes a constraint for TCR mobility. To define a link between [Ca^2+^]_i_ increase and actin cytoskeleton function, we used FACS analysis to measure F-actin (polymerized actin). PBL were either untreated or treated with increasing concentrations of ionomycin or latrunculin B (as a control of actin cytoskeleton de-polymerization) for 15 minutes at 37°C. Cells were fixed, permeablized, and stained with Alexa-488 labelled phalloidin to selectively tag F-actin. As shown in Figure 3, treatment with latrunculin B decreased the amount of F-actin in T cells in a dose-dependent fashion, supporting the notion that FACS analysis is suitable for quantification of F-actin [25]. Treatment of T cells with ionomycin induced a moderate dose-dependent increase in F-actin. These results indicate that [Ca^2+^]_i_ increase augments the amount of F-actin in T cells and suggest that the increase in actin polymerization could be responsible for the observed effect of [Ca^2+^]_i_ increase on TCR mobility.

### Modeling reveals effective TCR binding parameters to the actin cytoskeleton

We have shown that the observed decrease in TCR mobility following ionomycin treatment is mediated by the cortical actin cytoskeleton. We can extend the pure diffusion model, used to obtain the results in Table 1, to include binding interactions between TCR and the actin cytoskeleton as follows,
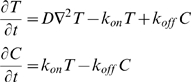
where T is the concentration of diffusing TCR, C is the concentration of TCR bound to the cytoskeleton, D is the diffusion coefficient of TCR, *k_off_* is the unbinding rate, and *k_on_* is the effective binding rate. The effective binding rate is *k_on_* = *k̅* *_on_S*, where *k̅* *_on_* is the intrinsic binding rate of TCR directly to the cytoskeleton or binding through a putative mediator that links TCR to the cytoskeleton. *S* is the concentration of binding sites available to diffusing TCR which are assumed to be homogenously distributed. This binding plus diffusion model has been previously used in FRAP analysis to extract binding parameters [26].

**Figure 4:**
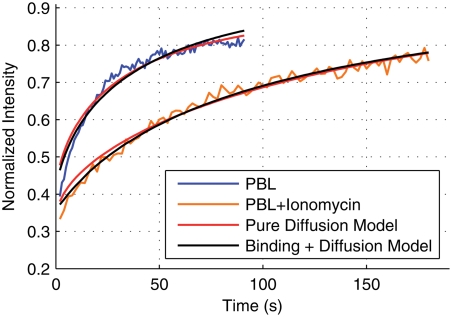
Fitting the diffusion and binding model to FRAP recovery curves. We show the fit of the pure diffusion model (red) and the binding+diffusion model (black) to the PBL data and to the ionomycin-treated PBL. Both models provide a good fit to the recovery curves but a statistical test reveals that the binding model is significantly better at explaining the FRAP recovery when the PBL are treated with ionomycin (Table 2).

In Table 2 we summarize the results of fitting the pure diffusion model and the binding plus diffusion model described above to the PBL data and the PBL+ionomycin data. When fitting the binding model the only free parameters are *k_on_* and *k_off_* and we set D = 0.0609 µm^2^/s, the free diffusion coefficient. The fitting procedure we used is described in Dushek et al [22]. We find that both the pure diffusion and binding models accurately fit both data sets (Figure 4). Using a statistical test to compare the explanatory power of each model (Akaike's Information Criterion, AIC) we find that both models explain the PBL data equally well. However, we find that the binding model has significant additional power in explaining the PBL+ionomycin data (compare the probabilities in Table 2). This suggests that TCR binding is quite possibly responsible for the slow FRAP recovery after ionomycin treatment as opposed to explanations involving a smaller mobile fraction or a change in the underlying TCR diffusivity. In this case, the binding model reveals rapid binding kinetics between the TCR and the putative cytoskeletal binding partner with *k_on_* = 0.22 s^−1^ and *k_off_* = 0.05 s^−1^. Put another way, in the experiments with PBL+ionomycin, a TCR might be expected to bind the cytoskeleton on average every 5 s and unbind after 20 s.

**Table 2:** Model fitting to FRAP recovery curves.

	PBL				PBL+Iono			
	SSR	AIC	ΔAIC	Prob.	SSR	AIC	ΔAIC	Prob.
Pure Diffusion Model	0.0449	−680.06	0.799	0.40	0.0281	−722.27	38.71	0.00
Diffusion+Binding Model	0.0445	−680.86	0.000	0.60	0.0183	−760.97	0.00	1.00

SSR – Sum of squared residuals, AIC – Akaike's Information Criterion, Prob – Probability that the model is the better descriptor of the FRAP recovery curve based on AIC.

## Discussion

In the present work we have shown, in agreement with previous studies [9], [10], that TCR are constitutively mobile on the surface of T cells. This lateral mobility (with a diffusion coefficient in the region of 0.05–0.1 µm^2^/s) is in principle sufficient to allow individual TCR to patrol the entire T cell surface within about 30 minutes. The diffusivity of TCR is similar in T cells at different stages of activation, indicating that this parameter is not involved in allowing activated T cells to be more responsive to antigenic stimulation.

Fast lateral mobility and rapid cell surface patrolling by TCR may be instrumental in resting conditions to allow T cells to rapidly sense antigenic stimuli. However it seems likely that, upon antigenic stimulation, regulated TCR motion would be needed to permit spatially localized signaling. Accordingly we find that [Ca^2+^]_i_ increase (a central and early signal in T cell activation) induces a significant decrease of TCR mobility. Our results also show that this reduction in TCR mobility requires a functional actin cytoskeleton. This suggests that following [Ca^2+^]_i_ increase, TCR become associated with actin cytoskeleton or with actin binding proteins. Fitting a model for TCR diffusion and binding to an immobile partner to our data suggests that this may indeed be the case.

Our observations, by showing that TCR may be bound or trapped by F-actin, raise the question of how they could move towards the IS. It is well established that F-actin is enriched at the IS [27]. Moreover, work from M.M. Davis and colleagues showed that cortical F-actin moves towards the T cell/APC contact site in antigens stimulated T cells [18]. Our present work points towards an important role of actin cytoskeleton in control of TCR motion although we did not focus in this study on TCR mobility during T cell/APC cognate interaction.

We also showed that pharamcological [Ca^2+^]_i_ increase results in a increased level of F-actin in T cells. The concentration of ionomycin at which a clear shift in F-actin cellular content was observed was 2.5 µg/ml. In the FRAP experiments, 0.5 µg/ml ionomycin (a concentration previously used to activate T cells, [16]) was sufficient to induce a reduction of TCR mobility. The reason for this discrepancy is presently elusive. It is possible that the effect on TCR mobility precedes significant actin polymerization. Alternatively, differences in sensitivity between the two experimental methods could be the reason for the observed discrepancy. This result suggests that upon initial conjugation of a T cell with a cognate APC the productive and localized engagement of a small number of TCR may lead rapidly via [Ca^2+^]_i_ increase and actin polymerization to global control of TCR motion.

Mathematical models of immune synapse formation commonly include TCR transport towards the center of the immune synapse, driven by the actin cytoskeleton [14], [28]–[31]. Important parameters in these models are effective reaction rates between TCR and the actin cytoskeleton, which to our knowledge have not been previously reported. For example, in the study of Burroughs et al., it was assumed that the effective on- and off-rates were identical and equal to 0.1 s^−1^. It would be interesting to investigate the effects of a larger on-rate and smaller off-rate, as reported in the present work, on the results of this and other models.

Several lines of evidence established a link between CAC polymerization and TCR signaling [32]. Treatment of T cells with drugs affecting actin cytoskeleton such as cytochalasin D, aborts antigen induced [Ca^2+^]_i_ increase indicating that actin polymerization is required to sustain signaling in T cells [16]. Moreover, deficiency or mutation in several molecules regulating the actin cytoskeleton such as Vav-1, Cdc42 and Wiskott-Aldrich syndrome protein (WASP) affect IS formation [33]. Some of these proteins such Nck and WASP are recruited to the TCR signaling area together with polymerized actin [34].

Interestingly, while actin cytoskeleton polymerization is usually considered to be required for the activation of calcium pathway [16], [32], here an original reverse mechanism is proposed: [Ca^2+^]_i_ increase is shown to enhance actin cytoskeleton polymerization and in turn modulate TCR mobility.

We speculate that the binding or trapping of TCR by the actin cytoskeleton network could also be instrumental in favoring the assembly of the TCR associated signaling cascade. The notion that TCR move very rapidly is difficult to reconcile with the necessity of assembling complexes of adaptor molecules and signaling components at the IS. The observation that TCR mobility is affected by global [Ca^2+^]_i_ increase is compatible with the formation of localized signaling scaffolds favoring complex molecular interactions. This hypothesis is in agreement with reported data by M. Dustin and colleagues showing that a functional actin cytoskeleton is required to allow TCR micro-clusters to signal and that high concentration of polymerized actin is confined to TCR clusters [13]. It is tempting to speculate that global [Ca^2+^]_i_ increase induced by the engagement of a few TCR at the T cell/APC contact site (via polimerization of CAC) might lead to the rapid formation of a much greater number of TCR clusters in the nascent IS. If so, this would equip T cells with pre-formed signaling units ideal for sensitive detection of a limited number of antigenic ligands.

Our work outlines a previously unexpected link between the calcium pathway, the actin cytoskeleton and TCR mobility. Although we have not examined the detailed mechanisms of this triangular interaction our results highlight the ability of TCR populations to control their own mobility via calcium signaling.

## Materials and Methods

### Cell isolation and culture

Purification of CD4^+^ human PBL: T cells were isolated from the whole blood of healthy volunteer donors (Centre de Transfusion Sanguine, CHU Purpan, Toulouse) as described [35]. Briefly, PBMC were purified from blood by centrifugation through Ficoll-Hypaque (Pharmacia Biotech, Sweden). CD4^+^ T lymphocytes were purified using RosetteSep Kit (StemCell Technologies, Vancouver, Canada). Cell purity was assessed by FACS analysis (Facscan, Becton Dickinson) using FITC-labelled anti-CD4 mAb (clone RPA-T4, BD Pharmingen). CD4^+^ fractions were routinely ∼90% pure. Before use, purified T cells were cultured in complete RPMI 1640 (Gibco, Paisley, Scotland) containing 5% human serum. In parallel experiments, umbilical Cord Blood T Lymphocytes from healthy volunteer donors (CBTL) were purified from whole cord blood (kindly provided by the Obstetric Service of the Hôpital Paule de Viguier, CHU Purpan, Toulouse) as above described. In some cases CBTL were cultured and expanded for 14 days in RPMI 1640, 5% human serum, IL-2 (150 IU/ml) using Dynabeads CD3/CD28 T Cell Expander (Dynalbiotech, Oslo, Norway). Bead: cell ratio was 1∶1.

### Fluorescence Recovery After Photobleaching

Anti-CD3ε mAbs (TR66, IgG1, [36] were digested by using the IgG1 Fab and F(ab)'2 kit (Pierce biotechnologyTM, Rockford). Fab fragments (5 mg/ml in 0.1 M Sodium carbonate, pH = 9.3) were labelled with Cy5 Monoreactive dye pack (Amersham BioscienceTM, Piscataway). The labelled Fab fragments were separated from non-conjugated dye by gel chromatography by the use of Nap-10 Columns (Amersham Bioscience). This step was followed by an overnight dialysis in 1× PBS by using a Lyser Dialysis cassettes (Pierce biotechnology) [7].

PBMC derived CD4^+^ T cells were washed and re-suspended in RPMI, 5% FCS, 10 mM Hepes. Cells were loaded with 2 µM Fluo4 AM (Molecular Probes, Leiden, The Netherlands) for 30 minutes at 37°C. After washing, cells were stained with 30 µg/ml CD3/Cy5 Fab in RPMI, 1% FCS at 4°C for 30 minutes. Cells were washed and kept in ice until used for FRAP experiments. FRAP was performed in Lab-Tek chambers (Nalgene Nunc, Rochester, NY) in pre-warmed 5% FCS/10 mM HEPES at 37°C, 5% CO_2_ on a confocal microscope (LSM 510; Carl Zeiss, Jena, Germany) using a Plan-Apochromate 63× objective and the 633 nm laser. A rectangular region (2 µm×1.4 µm) was defined on the surface of T cells. This region was irradiated using the 633 nm, 543 nm and 488 nm lasers with 100% intensity for 3 s. The aperture of the pinhole was adjusted to obtain optical slices of 2 µm depth. Before and after bleaching, the whole field was visualized by irradiating with 488 nm laser with 4% intensity and 633 nm laser with 17% intensity. Images were taken at 1 second intervals.

In some experiments, cells were treated either with ionomycin (0.5 µg/ml) at the time of the time-lapse recording. In some experiments cells were treated with cytochalasin D (10 µM) or latrunculin B (50 nM) before the beginning of the assay.

### Fitting procedures

We used the method for FRAP measurements of surface diffusion described in [37]. Briefly, a small region of the cell membrane was bleached and the fluorescence recovery curve was fit using a two-dimensional recovery equation to estimate the TCR diffusion constant and the mobile fraction of TCR. The Matlab function lsqcurvefit was used for fitting.

We also fit a simple exponential to every FRAP experiment. Using the time-constant of the recovery as an indicator of TCR mobility gave the same conclusions as using the diffusion coefficient (analysis not shown).

### Quantification of F-actin by phalloidin staining and flow cytometry

A method described by Downey et al [38] was used with some modifications [25]. Briefly, after incubation at 37°C with different draugs PBL were fixed with 3% PFA, permeabilized with 0.1% saponin and stained with 160 nM Alexa 488-labelled phalloidin. Cells were analyzed on a FACSCalibur (Becton Dickinson). Gating was done to exclude by forward and side scatter criteria cell debris and cell clumps. In some samples T cells were treated with 0.5–2.5 µg/mL ionomycin or with 50 to 500 nM latrunculin B for 15 minutes at 37°C, before fixation.
